# Cost-effectiveness of monitoring ocular hypertension based on a risk prediction tool

**DOI:** 10.1136/bmjophth-2024-001741

**Published:** 2024-08-28

**Authors:** Hangjian Wu, Gus Gazzard, Anthony King, James Morgan, David Wright, David P Crabb, Yemisi Takwoingi, Augusto Azuara-Blanco, Verity Watson, Rodolfo Hernández

**Affiliations:** 1 Health Economics Research Unit, Institute of Applied Health Sciences, University of Aberdeen, Aberdeen, UK; 2 NIHR Biomedical Research Centre, Moorfields Eye Hospital NHS Foundation Trust, London, UK; 3 Institute of Ophthalmology, University College London, London, UK; 4 NYU Langone Health, New York, New York, USA; 5 Department of Ophthalmology, Nottingham University Hospitals NHS Trust, Nottingham, UK; 6 Cardiff Centre for Vision Sciences, University of Wales College of Medicine, Cardiff, UK; 7 Centre for Public Health, Queen's University Belfast, Belfast, Northern Ireland, UK; 8 Division of Optometry & Visual Science, City University, London, UK; 9 Public Health, Epidemiology and Biostatistics, University of Birmingham, Birmingham, UK

**Keywords:** Glaucoma, Ocular Hypertension

## Abstract

**Background/Aims:**

To assess the cost-effectiveness of making treatment decisions for patients with ocular hypertension (OHT) based on a risk prediction (RP) tool in the United Kingdom.

**Methods:**

A discrete event simulation model was constructed to compare the cost-effectiveness of an alternative care pathway in which the treatment decision was guided by a validated RP tool in secondary care against decision-making based on the standard care (SC). Individual patient sampling was used. Patients diagnosed with OHT and with an intraocular pressure of 24 mm Hg or over entered the model with a set of predefined individual characteristics related to their risk of conversion to glaucoma. These characteristics were retrieved from electronic medical records (n=5740). Different stages of glaucoma were modelled following conversion to glaucoma.

**Results:**

Almost all (99%) patients were treated using the RP strategy, and less than half (47%) of the patients were treated using the SC strategy. The RP strategy produced higher cost but also higher quality-adjusted life years (QALYs) than the SC strategy. The RP strategy was cost-effective compared with the SC strategy in the base-case analysis, with an incremental cost-effectiveness ratio value of £11 522. The RP strategy had a 96% probability of being cost-effective under a £20 000 per QALY threshold.

**Conclusions:**

The use of an RP tool for the management of patients with OHT is likely to be cost-effective. However, the generalisability of the result might be limited due to the high-risk nature of this cohort and the specific RP threshold used in the study.

**WHAT IS ALREADY KNOWN ON THIS TOPIC**

- Despite the development and continuing validation of the Ocular Hypertension Study–European Glaucoma Prevention Study tool, one of the most credible risk prediction models for developing glaucoma, the cost-effectiveness of implementing such risk prediction tool in the NHS has rarely been discussed. The recent National Institute for Health and Care Excellence guideline highlighted the need for further research on risk prediction tools.

**WHAT THIS STUDY ADDS**

- We investigated the cost-effectiveness of making treatment decisions for ocular hypertensive patients based on a recently validated risk prediction tool using the electronic medical records of UK patients. We find that the risk prediction strategy produced higher costs and higher quality-adjusted life years (QALYs) than the standard care strategy. The risk prediction strategy was cost-effective in the base-case analysis under a £20 000 per QALY threshold and had a 96% probability of being cost-effective in probabilistic sensitivity analysis.

**HOW THIS STUDY MIGHT AFFECT RESEARCH, PRACTICE OR POLICY**

- The results suggest that managing ocular hypertensive patients using a risk prediction tool can be cost-effective depending on patients’ risk of conversion, the predictive power of the tool and the risk threshold used.

## Introduction

 Glaucoma is the second most common cause of irreversible registered blindness, affecting around 60 million of the world population and 10% of those aged 75 or above in the UK.^1 2^ Ocular hypertension (OHT) and early glaucoma are mostly asymptomatic but can result in lifetime visual impairment and blindness without proper treatment. Intraocular pressure (IOP) is the only modifiable risk factor for conversion to glaucoma and disease progression. Therefore, long-term routine monitoring and treatment of elevated IOP and visual field (VF) are key to controlling the disease and reducing the risk of visual impairment. OHT monitoring in the UK includes the assessment of IOP and signs of visual deterioration (eg, VF or optic nerve changes). Medical treatments such as prostaglandin analogues (PGAs) and/or beta-blockers (BB) lower IOP and help deter disease progression. If medical treatments fail, laser and surgery options exist for further management.

In the UK, patients with OHT are monitored either in primary care (eg, community optometrists) or secondary care (eg, eye hospital doctors). The stratification of patients across settings is based on a patient’s risk of developing lifetime visual impairment.^1^ In England, over one million glaucoma-related outpatient visits take place in secondary care eye services each year.^3^ Population ageing means that the number of OHT patients, suspected glaucoma patients and confirmed glaucoma patients can rise by 16%, 18% and 44% between 2015 and 2035, respectively.^4^ However, unnecessary referrals can overburden the NHS. The Royal College of Ophthalmologists’ Glaucoma Commissioning Guidance stated that many patients currently referred to secondary care can be discharged to primary care health professionals to free up secondary care NHS resources.^3^

An appropriate risk stratification tool using multiple clinical criteria to assign risk levels to individual patients can potentially release resource use in secondary care, yet there is no nationally agreed model for glaucoma management in the UK.^5^ Simple risk stratification tools primarily based on VF measures can be misleading, while tools with multiple criteria can be complex to implement.^5^ An RP model powered by multiple regression analysis is a promising candidate, as it incorporates multiple risk factors into the analysis and produces a simple risk estimate which facilitates its application. The glaucoma RP tool that has been developed and validated based on the results of the Ocular Hypertension Study (OHTS)^6^ and the European Glaucoma Prevention Study (EGPS) is the most credible one so far,^7^ yet it has not been recommended by clinical guidelines.^1^ The tool estimates the individual’s 5 year risk of conversion to glaucoma based on the following risk predictors: age, IOP, central corneal thickness (CCT), a measure of the VF test (pattern standard deviation [PSD]) and the optic nerve (the vertical cup to disc ratio; vCD ratio). The application of an RP tool with good predictive power could be used to identify patients who are most suitable to be monitored in primary care reducing demand on ophthalmology departments in secondary care and allowing health professionals in secondary care to focus on patients with a higher risk of vision loss.

Economic evaluations assess the relative efficiency of alternative healthcare technologies in terms of their cost and consequences.^8^ In the literature, most economic evaluation studies of OHT or glaucoma monitoring examine the cost-effectiveness of different monitoring frequencies or delegating care to appropriately trained primary care healthcare professionals compared with the usual care in secondary care.^9^,^11^ Only one study evaluated the cost-effectiveness of using a validated RP tool based on the OHTS–EGPS dataset to assist clinical decision-making.^12^ The authors used two non-UK-based clinical trial datasets and two small observational datasets to validate the RP tool. However, the new National Institute for Health and Care Excellence (NICE) guideline highlights the need for further research on RP tools.^1^ First, it has been 12 years since the publication of Burr *et al* (2012)’s work, during which time the NICE guidelines have been updated significantly (eg, the treatment prioritised for OHT patients and suggested intervals of clinical tests). New evidence in modelling disease progression has also emerged based on recently published articles.^13^ Second, new evidence shows that a new validated and calibrated RP tool using a large UK-based dataset from electronic medical records (EMRs) has a moderate improvement in predictive power compared with the previous RP tool based on the OHTS–EGPS dataset (information is available from the authors on request). In this study, we address these gaps by investigating the cost-effectiveness of this UK-based RP tool using a new decision analytic model.

## Methodology

### The model

**Figure 1:**
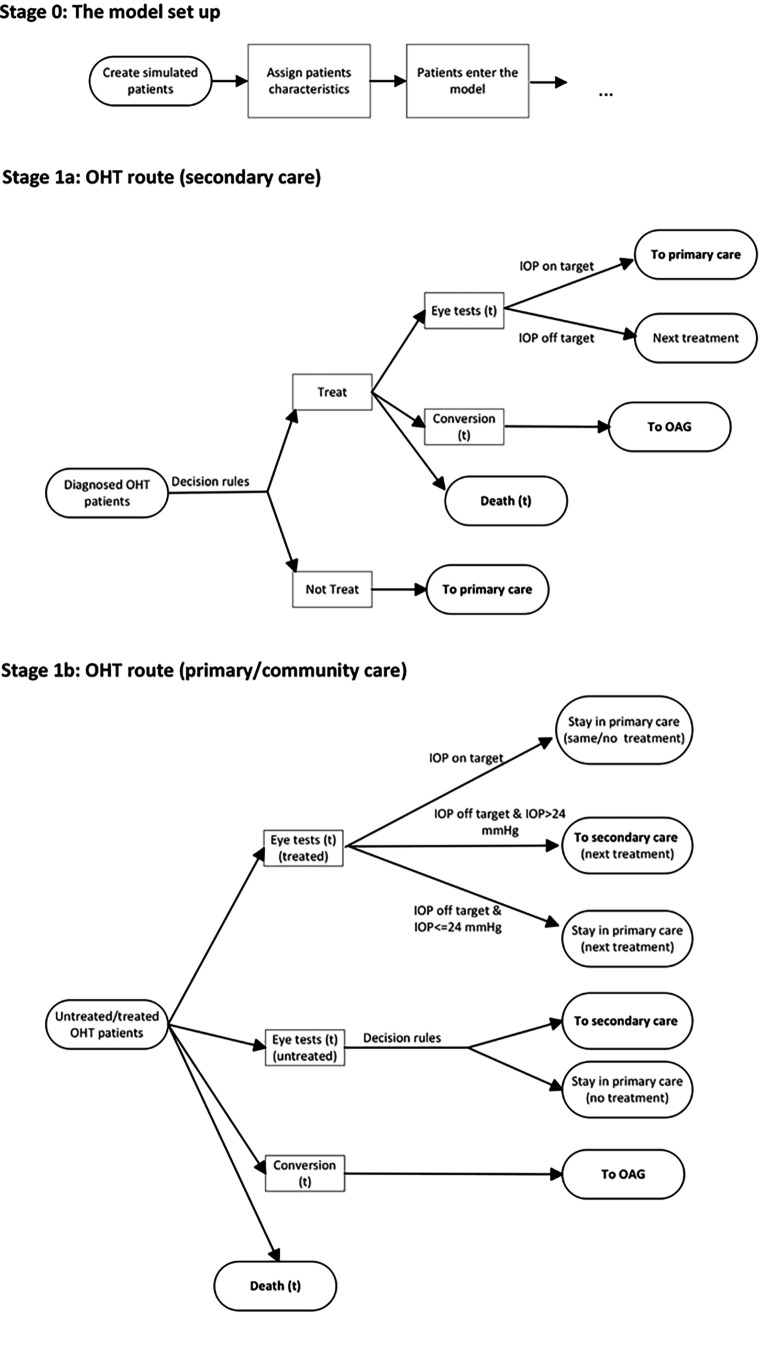
A schematic of the model structure. Diagnosed ocular hypertension (OHT) patients with intraocular pressure (IOP) of ≥24 mm Hg entered the model with a set of predefined individual characteristics related to their risk of conversion to glaucoma. An initial decision on treatment was made by a secondary-care health professional. Patients without treatment were referred for annual check-ups in primary care and can be referred back to secondary care following an unfavourable check-up. Patients who met the initial treatment rule in secondary care were treated with prostaglandin analogues (80% of them) or selective laser trabeculoplasty (20% of them). Treated patients with ‘on target’ IOP (ie, IOP reduced by 20% or more compared with the baseline IOP after treatment) were returned to primary care after one clinical visit for continued monitoring, while the treatment was escalated for ‘off-target’ patients following the treatment sequence. For treated or untreated patients monitored in the primary care settings, an observed conversion to glaucoma would trigger a referral to secondary care, and an immediate eye assessment was assumed to be conducted by the hospital ophthalmologists/optometrists to confirm the evidence of glaucoma. Patients with negative glaucoma assessment results would be referred back to primary care, and those with positive assessment results were remained in secondary care. In addition, treated OHT patients monitored in primary care with IOP measures deemed ‘off-target’ would be referred to secondary care. (t) means it’s a time-to-event.

A discrete event simulation (DES) model was developed to model OHT and glaucoma monitoring and treatment.^14^ DES models offer flexibility and the ability to explicitly evaluate monitoring frequency.^15^,^17^ Diagnosed OHT patients with IOP of ≥24 mm Hg entered the model with a set of predefined individual characteristics related to their risk of conversion to glaucoma (figures1  2). An initial decision on the treatment was made by a secondary-care health professional (eg, a hospital ophthalmologist/optometrist). Patients without treatment were referred for annual check-ups in primary care. Patients who met the initial treatment rule in secondary care were treated with PGAs (80%) or selective laser trabeculoplasty (SLT) (20%).

**Figure 2:**
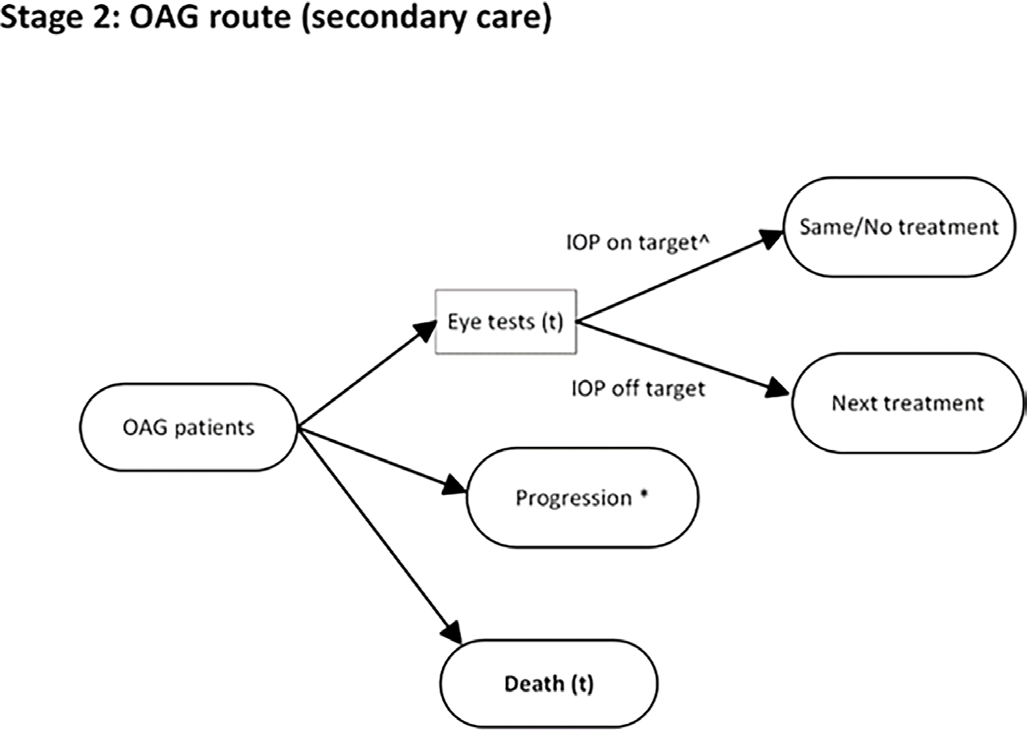
A schematic of the model structure. Confirmed glaucoma patients would be maintained in secondary care for regular eye assessment by the hospital ophthalmologists/optometrists. Patients with ‘on-target’ IOP would be continuously treated with the current treatment (or no treatment), while the treatment was escalated for ‘off-target’ patients following the treatment sequence. *(t)* means a time-to-event. *Progression to the next level of glaucomatous stage, which can be moderate, severe or visual impairment. Patients cannot progress further on reaching visual impairment. ^∧^‘on-target’ IOP means IOP reduced by 20% or more compared with the baseline IOP after treatment.

Throughout the model, patients repeatedly faced three ‘competing’ events: check-ups (eye tests), conversion to glaucoma (or progression to more advanced glaucoma for open-angle glaucoma [OAG] patients) or death, whichever option had the shortest time-to-event would occur next. The likelihood of the occurrence of these events was governed by the time-to-event values, which were based on patients’ characteristics and history of monitoring and treatment. Time-to-event was recalculated each time an event occurred. A schematic of the DES simulation is shown in figures1  2.

A population of newly diagnosed OHT patients with IOP of ≥24 mm Hg were simulated according to a set of predefined individual characteristics linked to their risk of conversion to glaucoma (ie, age, IOP, CCT, vCD ratio and PSD).^7^ Additional risk factors (ie, whether an individual has hypertension, family history of glaucoma, diabetes and biological gender) relevant to the RP tool were also included. Sampling was based on individual patient data extracted from the EMR dataset of the UK OHT patients. The mortality rate of the UK general population is sourced from the UK life table.^18^ Table 1 shows the detailed statistics of the individual characteristics.

**Table 1:** Baseline characteristics of the extracted individual patients

Baseline variables	Mean	SD	Data source
Number of individual patients in the extracted dataset	5740		
Age (years)	62.01	10.56	The EMR dataset (information is available from the authors on request)
CCT (μm)	558.66	35.83	
IOP (mmHg)	26.51	2.13	
PSD (dB)	1.63	0.34	
vCD ratio	0.46	0.17	
Hypertension (Y/N)	0.12	0.33	
Family history of glaucoma (Y/N)	0.26	0.44	
Diabetes (Y/N)	0.14	0.34	
Male (Y/N)	0.43	0.50	
Previously treated (Y/N)	0.36	0.48	
Mean deviation at conversion*	−2.94	2.67	
Life expectancy	Various		UK interim life tables 2018–2020 (gender average)^18^

* The mean deviations (MDs) at conversion were drawn from a gamma distribution with mean and SD extracted from the dataset. Individual patient sampling was not used due to missing data.

CCTcentral corneal thicknessIOPintraocular pressurePSDpattern standard deviationvCDvertical cup-to-disc

Disease progression is modelled by considering the time it takes to reach each disease state. The time-to-conversion to glaucoma for OHT and time-to-progression for OAG patients were estimated following van Gestel, Severens and Webers *et al* (2010)’s approach.^13^ Time-to-conversion was calculated based on patients’ current IOP, age and other relevant risk factors. A key VF outcome, mean deviations (MD), was used to represent glaucoma progression, which was assumed to be positively associated with patients’ IOP levels. The detailed calculation of time-to-conversion and time-to-progression can be found in online supplemental materials A1. A common glaucoma staging system was used to classify the VF outcome following Mills *et al* (2006).^19^ Online supplemental table A5 in online supplemental materials A1 provides details of the glaucoma stages and corresponding MD values.

The clinical pathways, treatment sequence and eye test intervals for OHT and glaucoma monitoring were developed based on the 2022 NICE guidelines^1^ and the advice of experts, consisting of four ophthalmologists, two health economists and two statisticians. Patients or the public were involved in the design, conduct, reporting or dissemination plans of our research. Two pathways were considered:

- OHT monitoring based on standard care (SC).
- OHT monitoring based on an RP tool.

All pathways are comprised of both primary care and secondary care monitoring and treatment but differ in the criteria for accepting patients for treatment. For the SC pathway (comparator), the criteria for accepting patients for treatment in secondary care were discussed in several meetings with the clinicians in the project management group, and a decision table was created based on the level of IOP, age and the patient’s central corneal thickness (CCT) (see online supplemental table A2).

For the RP pathway (intervention), it was assumed that the RP tool was used by hospital ophthalmologists/optometrists to make clinical decisions regarding the treatment in secondary care. The RP tool was developed and validated using a large UK-based dataset retrieved from the EMRs (information is available from the authors on request). The RP tool provided risk estimates of the 5 year risk of conversion to glaucoma used to inform the treatment decision. Based on expert views, patients with a 5 year risk of conversion of ≥6% were initially treated in secondary care and remained in primary care without treatment otherwise. Additional explanations are provided in online supplemental material A1.

A common treatment sequence was developed based on the NICE guidelines and expert views. Treatment effectiveness data were obtained from various sources in the literature.^1120^,^24^ The treatment sequence and effectiveness were detailed in online supplemental material A1.

The unit costs for monitoring were obtained from the NHS reference cost and Department of Health (NHS sight test fee).^25 26^ Medications and surgical treatments were valued using national unit cost sources and validated trial studies.^25^,^27^ We used the EQ-5D to value quality of life for each disease state in the model (ie, OHT, mild, moderate, severe glaucoma and visual impairment) based on a valuation study of an OAG population from the UK.^28 29^ Clinical effectiveness, costs and utilities are reported in table 2. Additional explanations are provided in online supplemental material A1.

**Table 2:** Parameters and sources for the treatment effectiveness, costs and utilities

	Data input	Data source
Treatment		
PGAs (Latanoprost)*	Mean: 0.29SD: 0.08	Valk *et al* (2005)^20^ and van Gestel (2012)
PGAs and BB (Latanoprost and Timolol; additional effectiveness compared with Latanoprost)*	Mean: 0.14SD:0.08	van Gestel (2012)^21^ and Webers *et al* (2008)^22^
SLT	Mean: 0.312SD: 0.08	Mean estimate: Chi *et al* (2020)^23^; SD: assumption
Trabeculectomy	Mean: 0.447SD: 0.189	Kirwan *et al* (2013)^24^ and Crabb *et al* (2014)^11^
Costs for monitoring†		
Secondary care: IOP only	£147	NHS reference costs (2021–2022)^25^; Ophthalmology outpatient attendance (service code: 130)
Secondary care: IOP and VF	£294	Assumption. Twice the unit cost for IOP only
Primary care: NHS sight test fee: IOP only	£11.57	Assumption. Half the unit cost for IOP and VF test fee
Primary care: NHS sight test fee: IOP and VF	£23.14	Department of Health (General Ophthalmic Services: NHS sight test fee, updated in April 2023)^26^
Costs for treatments†		
Latanoprost	£149.76 per year with 2.5 mL = £12.48	BNF 2023; Xalatan
Latanoprost and Timolol	£171.84 per year with 2.5 mL = £14.32	BNF 2023; Xalacom
SLT	£151 per patient	Gazzard *et al* (2019)^27^
Trabeculotomy	£1694 per patient	NHS reference costs (2021–2022); glaucoma surgical procedures (HRGs code: BZ92B; average of total cases)
Disease states		
Patients with OHT	0.8015	Assumption
Patients with mild OAG	0.8015	Burr, Kilonzo, *et al *(2007)^28^
Patients with moderate OAG	0.7471	Burr, Kilonzo, *et al* (2007)^28^
Patients with severe OAG	0.7133	Burr, Kilonzo, *et al* (2007)^28^
Visually impaired OAG patients	0.535	Burr, Mowatt, Hernández, *et al* (2007)^29^

* Assuming one bottle of the eyedrops per month per patient

† The cost for latanoprost and timolol were used to cost the PGAs and BB medical treatment, respectively. These unit costs were obtained from the British National Formulary (BNF), assuming one bottle of the eyedrops per month per patient. Unit cost for the Trabeculectomy was obtained from the NHS reference costs. The unit cost for the SLT was obtained from the LiGHT trial.

BBbeta-blockersIOPintraocular pressureOAGopen-angle glaucomaOHTocular hypertensionPGAsprostaglandin analoguesSLTselective laser trabeculoplastyVFvisual field

### Data analysis

A cohort of 50 000 patients with diagnosed OHT were used in the simulation using Treeage (2023 R2.0) for the base-case analysis (the model is available from the authors on request). All analyses were based on the NHS perspective with all costs expressed in GBP and 2021/2022 UK prices. The adjustment was conducted using a web-based tool.^30^ The time horizon of the model was lifetime with cost and utilities discounted at an annual rate of 3.5%.

To identify the key drivers of uncertainty around the costs and effectiveness, one-way and probabilistic sensitivity analyses (PSA) were conducted for (a) the threshold of treatment decision regarding the RP strategy, (b) medication and monitoring costs and (c) adherence rate to medication. The high number of simulated patients (eg, 50 000) increased the model running time but, on visual inspection, produced similar results to those obtained for 10 000 simulated patients. Therefore, 10 000 simulated patients with 1000 replications (second-order uncertainty) were used for sensitivity analyses.

### Model validation and calibration

The model has been carefully validated based on the internal dataset used and several external data sources, with several calibrations being made. Details can be found in online supplemental material A2. A health analysis plan is available on request.

## Results

### Base-case analysis

The simulated results for the base-case scenarios are shown in table 3. Almost all (99%) patients were treated in the RP strategy, while about 47% of patients were treated in the SC strategy. For the SC and RP strategies, 57% and 53% of the patients were estimated to have converted to glaucoma, respectively. In the SC strategy, more patients progressed to moderate (24%) and severe (11%) glaucoma and visual impairment (5%), which implied quality-adjusted life year (QALY) losses due to VF defects. This was not surprising as more patients received treatment in the RP strategy. Regarding cost-effectiveness, the RP strategy incurred higher costs but gained higher QALYs than the SC strategy. The difference in QALYs between strategies was relatively small as the strategies differed mainly in the decision to treat determined at the start of the model. The RP strategy was cost-effective compared to the SC strategy with an incremental cost-effectiveness ratio (ICER) (£11 522) which was below the cost per QALY threshold of £20 000 used by NICE.

**Table 3:** Cost-effectiveness results for the base-case analysis

Pathway	Proportion of patients initially treated (%)	Proportion of patients in each state at the end of model run (%)				
		OHT	OAG mild	OAG moderate	OAG severe	Visual impairment
Standard care strategy	47%	43%	17%	24%	11%	5%
Risk prediction strategy	99%	47%	17%	22%	10%	4%
	**Average total cost (£**)	**Incremental cost (£**)	**Average total QALYs**	**Incremental QALYs**	**ICER (£**)	
Standard care strategy	4662		10.89			
Risk prediction strategy	4925	262	10.92	0.023	11 522	

Proportion of patients who were initially allocated to treatment based on the decision algorithm

ICERincremental cost-effectiveness ratioQALYquality-adjusted life year

### One-way sensitivity analysis

Overall, the RP remained cost-effective when the adherence rate was decreased to 75%, the cost of medication increased by up to 50% or the cost of monitoring increased by up to 50%. However, the change of the risk threshold for the RP tool had the largest impact on the ICER—the RP strategy became less cost-effective as the threshold increased, and ICER exceeded the cost-effective threshold of £20 000 when the risk threshold was more than 12%. The impact of medication costs is generally larger than the one for the monitoring costs. For example, increasing the cost of PGA up to 50% raise the ICER value from £12 100 up to £18 076 (ie, a 49% increase), while the cost of primary care full test up to 50% raise the ICER value from £12 100 up to £13 137 (ie, an 8.5% increase). The full sensitivity analysis results are presented in online supplemental material A3.

### PSA

The cost-effectiveness scatterplots and cost-effectiveness acceptability curves can be found in online supplemental figures A2 and A3 in online supplemental material A3. The results showed that the RP strategy had a 98% probability of being cost-effective at the £20 000 per QALY threshold, which was consistent with the base-case results.

## Discussion

This study investigated the cost-effectiveness of an RP tool used in making clinical decisions in OHT monitoring. The costs and effectiveness of an RP tool used by health professionals were examined against the SC pathway using a DES model. Our results demonstrate that making treatment decisions based on our RP tool used in a secondary care setting can be cost-effective. This conclusion remains qualitatively unchanged against different scenarios and sensitivity analyses, except for a change in the risk threshold used to decide on treatment initiation. For a 5 year risk of conversion to glaucoma threshold of 12% or above, the RP strategy stopped being cost-effective.

A similar UK-based study concerning OHT monitoring was conducted by Burr *et al* (2012) in which the cost-effectiveness of two RP strategies were compared against a ‘treat-all’ strategy in which all patients were offered medication with no active monitoring of conversion.^12^ The RP strategies in their study were not considered cost-effective using a £30 000 per QALY threshold. The discrepancy in findings is not surprising, as the model settings in our study have been tailored to reflect the current NICE guidelines and updated knowledge on modelling time to conversion and progression. We also had access to a comprehensive patient-level dataset extracted from EMRs, which allows us to perform individual patient sampling. In our study, the cohort had a higher 5 year risk of conversion compared with the simulated cohort in Burr *et al* (2012) (ie, 17% vs 10% patients converted to glaucoma in 5 years). Another notable difference is the use of a calibrated RP tool based on the patient records of UK OHT patients. Some US-based studies suggested that treating high-risk cohorts, such as those with advancing age, higher IOP, thinner CCT or with a 5 year risk of conversion of 10% or higher (based on the OHTS RP tool), against a ‘treat-all’ or ‘treat-none’ strategy, were likely to be cost-effective, which was inconsistent with our results.^31 32^

The strategies compared in this study differ only in the decision algorithm used to determine whether to offer treatment with the RP strategy under the current risk threshold, indicating a very high proportion of patients being initially treated with medications or SLT. The findings imply that medications and SLT are inexpensive, safe and effective treatment options that delay conversion to glaucoma and glaucoma progression, especially for a high-risk cohort such as the sample used in this study. This result is consistent with findings from the OHTS trial in which high-risk OHT patients benefited the most from the treatment.^33^ However, the message cannot be simply interpreted as ‘treating more people is always cost-effective’ since several factors need to be considered in the implementation of clinical practice: (a) our sample includes a large proportion of patients with high risk profiles; in reality, more low-risk patients would need to be discharged to primary care for regular monitoring without treatment and (b) patient-centred care has been an important aspect of OHT and glaucoma treatment in the UK. Treatment decisions must be tailored based on individual patient needs and take into account factors such as eyedrop tolerance and adverse effects.^34 35^ Patients with intolerance to eyedrops and no immediate risk of conversion to glaucoma may not be offered treatment.

This study used a large-scale UK-based dataset extracted from the EMRs to model patient characteristics and adopted a comprehensive modelling approach, which reflects the current advances in disease progression modelling and updated NICE guidelines. This study also has three limitations. First, the RP tool used has limited predictive power with a concordance index (ie, c-index) of 0.69 in a recent validation study using UK OHT patients, while c-index of 1 represents a perfect prediction (information is available from the authors on request). Therefore, the cost-effective results of the RP strategy might be due to the particularly high-risk cohort defined in the model and the specific threshold used that result in almost all patients being treated in the RP strategy. The RP tool seems to fail to discriminate between those who need treatment and those who do not when the risk threshold for treatment is raised, which partly explains the inconsistency between the results of this and Kymes *et al* (2006)’s study.^32^ Second, the risk stratification threshold (ie, 6%) used in this study is only based on one study (ie, Kass *et al* (2010)^33^ and has not been widely discussed in the literature. However, our sensitivity analysis results show that the risk threshold can be a key factor affecting the cost-effective results. Third, we attached a zero R&D and production cost to the RP tool based on the assumption that these costs would be less important in the long run. However, little is known about the operating costs of using the risk calculator in clinical practice. Studies that investigate the monitoring of chronic conditions using digital technology suggest that operating costs such as integration and training costs may be nonnegligible.^36^ Our results suggest that further studies are needed to confirm the observed cost-effectiveness analyses of monitoring strategies based on a more advanced RP algorithm, and the economic evaluation should incorporate fixed and running costs of applying the RP tool.

## Conclusion

In conclusion, NICE has recommended the development of the RP algorithm for developing glaucoma in its recent guidance. Based on a recently validated RP tool using a UK-based dataset, we investigated the cost-effectiveness of using this tool to guide treatment decision in a secondary care setting compared with the SC. The results show that the RP tool is likely to be cost-effective, although this is subject to limitations regarding the characteristics of the sample used and the discriminatory power of the risk tool. Future research can extend the analysis to incorporate improved tools and different populations.

## supplementary material

10.1136/bmjophth-2024-001741 online supplemental file 1

## Data Availability

Data are available upon reasonable request. Data may be obtained from a third party and are not publicly available.
