# Supplementary material for: Cost-effectiveness of monitoring ocular hypertension based on a risk prediction tool
Source: BMJ Open Ophthalmol. 2024 Aug 28;9(1):e001741. doi: 10.1136/bmjophth-2024-001741 (PMC11367344; doi:10.1136/bmjophth-2024-001741)
Supplement: online supplemental file 1 [file bmjophth-9-1-s001.pdf]

Supplementary Material A1: additional description of model structure and data inputs

1. Discrete event simulation

A Discrete event simulation (DES) usually includes the following components: entities, attributes, events, relationships and outcomes. In this model, entities are simulated patients with diagnosed ocular hypertension (OHT) or open-angle glaucoma (OAG). Attributes are patients' characteristics, including age, intraocular pressure (IOP), and other risk factors of converting to glaucoma or progressing to more advanced glaucoma; events are eye tests (e.g., visual field (VF) and IOP tests), treatment, conversion to glaucoma (OHT only), progression to more advanced glaucoma states (OAG only), and death. Relationships are mathematical or logical relationships linking different elements together such as the mathematical expression linking the rate of disease progression with a patient's IOP level. Finally, outcomes include both clinical outcomes of interest (e.g., proportion of patients developing glaucoma) as well as economic outcomes (e.g., incremental cost, Quality-adjusted life years (QALYs) and Incremental Cost-Effectiveness Ratios (ICERs)).

2. Care pathways

(1) Referral, monitoring and treatment criteria for each pathway

Table A1: Referral, monitoring and treatment criteria for each pathway

Pathway	Referral criteria	Monitoring criteria	Treatment
SC	NICE guidelines and expert views (see the decision table A2 in supplementary material).<br><br>Patients maintained in primary care would only be referred to secondary care if (a) conversion to OAG being observed, or (b) untreated patients met decision rules, or (c) observed "off-target" during the last checkup and IOP above 24mmHg for the treated patients	Based on NICE guidelines and expert views (see the frequency tables in Table A6, supplementary material).	Those who met the treatment criteria were treated and kept in secondary care. 80% were initially treated with PGAs and the rest (20%) were treated with SLT. Those who did not meet the treatment criteria were maintained in primary care without treatment.
RP	Those of low risk (5-year risk of conversion<6%) were NOT treated and maintained at the primary care, but those of intermediate risk (5-year risk of conversion between 6-13%) or high risk (5-year risk of conversion>13%) were treated at secondary care.	Same as above.	Those who were treated followed the same treatment sequence as those in the SC pathway.

(2) Decision table for receiving treatment in the standard care pathway

Table A2: Decision table for receiving treatment in the standard care pathway

	D1	D2	D3	D4	D5	D6
**Conditions**						
IOP (mmHg)	>30	27-29	27-29	24-26	24-26	24-26
Age (year)						<50
CCT (um)		<600		<500	500-600	>600
Family history (Y/N)			Y		Y	Y

Notes: D1 means decision rule 1; family history is also drawn from the same multinomial distribution as baseline risk factors such as IOP, age and CCT, with the parameters describing the mean, SD and correlations extracting from the EMRs dataset.

(3) Risk stratification and calculation

The RP tool was developed and validated using a large UK-based dataset retrieved from the EMRs, comprising over 9,000 OHT patients from 11 hospital eye services in the UK with at least five years of follow-up.[1] The RP tool provided risk estimates of the 5-year risk of conversion to glaucoma used to inform the treatment decision; the calculation of the risk estimates is detailed below. Following Burr et al. (2012)'s risk classification[1], patients were split into three groups based on the risk estimates: low risk (<6%), intermediate risk (6-13%) and high risk (>13%). Based on expert views, the high and intermediate risk groups were initially treated in secondary care and the low-risk group remained in primary care for regular eye check-ups without treatment. However, low-risk patients could be referred to secondary care when their risk of conversion exceeded the predefined threshold (6%).

The risk estimates for the RP tool can be calculated in Equation A.1-A.2:

Risk estimate = $1 - 0.784 * e^{PI}$ (EQ A.1)

where $PI = 0.282 * (Age - 6.262) - 0.008 * (IOP - 24.731) + 0.058 * (CCT + 14.098) + 0.232 * (PSD - 8.379) + 0.099 * (vCD - 4.782) - 0.207 * hypertension - 0.026 * family\ history + 0.239 * diabetes - 0.036 * sex$ (EQ A.2)

where updated age and IOP, and the baseline data were used for these variables. Hypertension, family history, and diabetes are binary variables representing whether an individual has hypertension, family history of glaucoma or diabetes, respectively. The inclusion of these variables represented the effects of comorbidity on risk of conversion. Sex is a binary variable representing individual's biological gender (i.e., male or female). For example, a patient X with the baseline characteristics shown in Table A3 is estimated to have 10.1% of converting to glaucoma in the next 5 years.

Table A3: The profile of patient X

Age	Mean IOP	Mean CCT	Mean PSD	Mean vCDR	Sex	Family history	Diabetes	Hypertension
43.6	28	534	1.5	0.4	male	Yes	No	No

[1] Information is available from the authors upon request

(4) Assumption of the time needed for patient discharge

In the model, we assumed that stable OHT patients were discharged to the primary care only after one clinical visit. NICE guidelines suggested discharging patients 3-5 years after being stable in secondary care. However, several clinicians confirmed that the most common clinical practice is to discharge patients after one clinical visit due to capacity issues in UK hospitals.

3. Modelling time-to-conversion and time-to-progression

(1) The calculation of time-to-conversion

The survival function to conversion can be calculated by as Equation A3:

$$P = 1 - S = 1 - e^{-h_{it} * t} \qquad \text{(EQ A3)}$$

where $$h_{it} = HR_{AGE}^{\frac{(AGE_{it} - \overline{AGE_{ref}})}{10}} * HR_{IOP}^{(IOP_{it} - \overline{IOP_{ref}})} * HR_{OTHER_i} * h \qquad \text{(EQ A4)}$$

In Equation A3, P is the cumulative probability of conversion; S is the survival function; h_{it} is the current hazard rate of individual *i* at current event (time *t)*. In equation $A4, HR_{AGE}, HR_{IOP}, HR_{OTHER}$ are the hazard ratios of age (per 10 years older), IOP (per mmHg higher) and a combination of other risk factors (i.e., CCT, vCD and PSD), respectively; AGE_{it} is the age of individual *i* at current event *t*; IOP_{it} is the IOP of individual *i* at current event t; $\overline{AGE_{ref}}$ and $\overline{IOP_{ref}}$ are the average age and IOP of the referenced population of the OHTS-EGPS study;[2] h_{ref} is the calibrated hazard rate of the referenced population, which equals to 0.03.

Time-to-conversion estimates at event level can be derived from the equations above. A random draw from a uniform distribution is then used to determine the time-to-conversion value from the cumulative probability of conversion (i.e., only one probability was drawn for each patient at start of the model). As we sampled individual patients from the EMRs dataset, the conversion time for those who have been treated before the observation period may have been delayed compared with those who haven't received any treatment. To ensure a consistent starting point, the time-to-conversion for

the patients who have received treatment before was increased by an additional 2.7 years, representing an average effect of medications on time-to-conversion, extracted from Kass et al. (2010).[3]

(2) The calculation of time-to-progression

The current mean deviation (MD) score was modelled as the baseline MD plus the amount of MD decreased since conversion (Equation A5). Note that the MD values theoretically cannot increase due to the irreversible nature of glaucoma.

$$MD_{it} = MD\_Base_i - MDR_{it} * (T_t - T_{t-1})) \qquad \text{(EQ A5)}$$

where MD_{it} is the MD for individual *i* at time *t* (current time), which is assumed to be smaller than 0; MD_Base_i is the baseline MD; MDR_{it} is the progression rate of the MD; $T_t - T_{t-1}$ represents the current time minus the last time when progression was internally checked. Following van Gestel, Severens & Webers et al. (2010) approach,[4] progression rate was modelled as a function of current IOP. The higher the IOP, the faster the disease would progress. The progression rate of MD was calculated as Equation A6:

$$MDR_{it} = MDR_{ref} * HR_{it} = MDR_{ref} * HR_{IOP(OAG)}{}^{(IOP_{it} - \overline{IOP_{ref(OAG)}})} * HR_{OTHER} \qquad \text{(EQ A6)}$$

where MDR_{ref} is the average progression rate of MD referenced to the OAG population in the EMGT study;[5] $HR_{IOP(OAG)}$ is the hazard ratio of IOP (per 1 mmHg higher than average IOP in the referenced OAG population in the EMGT study); $\overline{IOP_{ref(OAG)}}$ is the average IOP level referenced from the OAG population in the EMGT study, which equals to 15.5 mmHg.[5]

Regarding the modelling of OAG progression, we mainly referenced the OAG population in the EMGT study, as the EMRs dataset contains insufficient information about the characteristics of OHT patients after converting to OAG. MDR_{ref} was drawn from a gamma distribution at patient level based on the empirical results extracted from van Gestel (2012).[6] Hazard ratios and average value for the IOP were

also extracted from the EMGT study. Table A4 shows the parameters used to calculate time-to-conversion and progression.

(3) Internal time-to-progression checks

OAG progression was checked internally with a fixed frequency (i.e., every 3 months) throughout the model after patients converted to OAG. This avoided failure in the detection of disease progression in time when time intervals between two clinical eye check-ups were large. Defined as a competing time event against time-to-death and time-to eye checkup, the internal checkup calculated MD with no implications on cost. The internal check-ups were not applicable to severe glaucoma patients, as the actual eye check-ups for them were assumed to be sufficiently frequent to identify progression. Table A4 shows the parameters used for the calculation of time-to-conversion and time-to-progression. Table A5 shows the glaucoma staging system used in this study.

Table A4: Parameters used to calculate time-to-conversion and progression

	**Parameters**	**Source**
**Hazard ratios**		
Age (decade)	1.26	EMGT
IOP (OHT) (mmHG)	1.09	EMGT
IOP (OAG) (mmHG)	1.13	EMGT
HR_{OTHER}	Ln(Normal(0,0.7))	EMGT and van Gestel (2012)[6]
**Average values of risk factors**		
Age (years)	55	EMGT
IOP (OHT) (mmHg)	24	EMGT
IOP (OAG) (mmHg)	15.5	EMGT
**Hazard rate in referenced OHT population**		
h	0.03	Van Gestel (2012)[6] before calibration
**Progression rate**		
Progression rate of mean deviation (reference)	Gamma (2, 0.014)	Van Gestel (2012)[6]

Table A5: Glaucomatous staging system based on Mills et al. (2006)

**Glaucoma severity**	**Mean deviation scores (dB)**
Mild	–0.01 to –6.00
Moderate	–6.01 to –12.00
Severe	–12.01 to –20.00
Visual impairment	≤-20.01

(4) The IOP level at any time point

The level of IOP is a key risk factor affecting both conversion and progression. Generally, a lower level of IOP compared with the baseline would delay the time-to-conversion and time-to-progression, and vice versa. We adopted the approach detailed in van Gestel (2012),[6] in which the IOP level at any point was modelled as the baseline IOP, plus an annual natural increase (i.e., 0.5%) and plus the IOP reduction due to any effective treatment.

4. Treatment effects

4.1 Treatment sequence

80% of the OHT patients were initially treated with PGAs and 20% treated with Selective laser trabeculoplasty (SLT). Recent development in the NICE guidelines suggests SLT being the initial treatment for those with OHT who had risk of blindness in their lifetime. However, we assumed (based on expert views) that only 20% of the OHT patients would go through SLT as a first treatment given the capacity restrictions in many UK hospitals. We assumed that SLT would not be repeated within 2 years based on the findings of the numbers of SLT from Gazzard et al. (2019).[7] SLT repeated more than 2 times during lifetime was possible only if the relative effectiveness of the SLT (compared with baseline IOP) was over 20%.

The next treatment following PGAs was a combination of PGAs and BB, which was then followed by SLT. Treatment escalation was triggered if a patient’s IOP was “off target” (defined as a baseline IOP reduction of less than 20%) or conversion to OAG was observed. A similar treatment sequence was assumed for patients converting converted to glaucoma, except that trabeculectomy was considered as a last resort if a patient did not meet the requirements for a SLT treatment. Patients were closely monitored without treatment after a SLT or trabeculectomy had been conducted, until a treatment escalation was triggered. The treatment sequence is visualised in Figure A1.

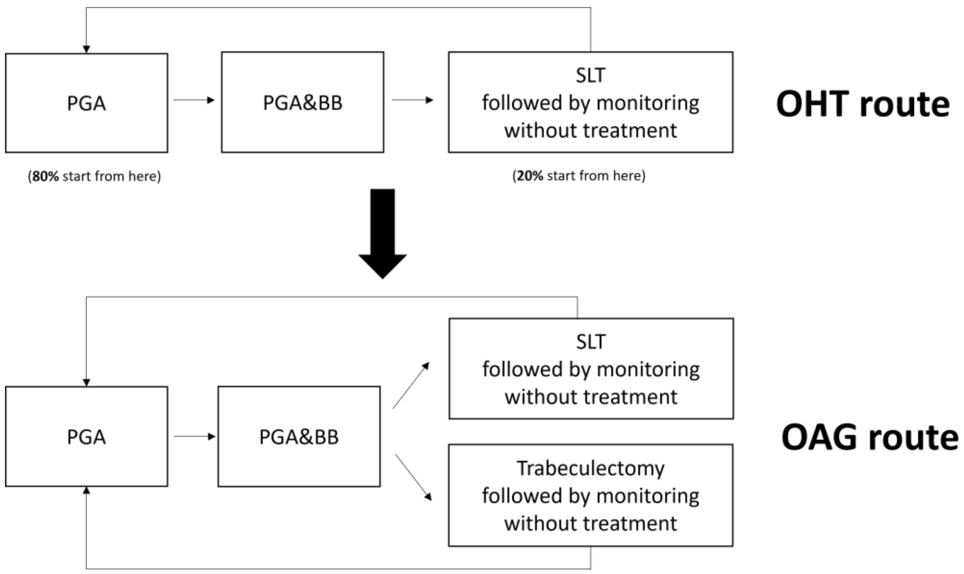

Figure A1: Treatment sequence for the ocular hypertension and open-angle glaucoma pathway.

4.2 Treatment effectiveness

(1) The effectiveness of SLT

The mean of the distribution describing the effectiveness of SLT was extracted from the results of a meta-analysis conducted by Chi et al. (2020)[8], and the SD was based on the assumption.

(2) The effectiveness of trabeculectomy

Following several prospective studies on the effectiveness of trabeculectomy, the number of trabeculectomies a patient can receive was restricted to one, and only a 9.4% of those who received trabeculectomy before were allowed to have a second trabeculectomy in their lifetime if needed.[9,10] The mean effectiveness of the trabeculotomy was initially extracted from the results of a literature review conducted by Crabb et al. (2014).[11] We later fitted a PERT distribution so that about 13% of the patients who received a trabeculotomy carried an effectiveness of less than 20% based on Kirwan et al. (2013).[10]

(3) The effectiveness of medication

The effectiveness of the first-line medication, i.e., PGAs, was extracted from van Gestel (2012)[6] who initially extracted the parameters from a meta-analysis study conducted by Valk et al. (2005),[12] and

then fine-tuned the effectiveness distribution based on views of clinical experts. The effectiveness of PGAs & BB was expressed in addition to the first-line drug. The parameters for the effectiveness distribution were extracted from van Gestel (2012)[6] who initially extracted the parameters from a systematic review study conducted by Webers et al. (2008),[13] and then fine-tuned the distribution based on views of clinical experts.

5. Frequency of clinical visits and precision of the measurement of progression to open-angle glaucoma

(1) precision of the measurement of progression to open-angle glaucoma

To reflect imperfect diagnostic accuracy of conversion from OHT to mild OAG in the community optometrists setting, sensitivity and specificity were assumed to be less than 1 (0.76 and 0.93 for sensitivity and specificity).[14] Perfect information for the diagnosis of glaucoma as well as the detection of disease progression was assumed in the secondary care setting (sensitivity and specificity of both conversion and progression equal to 1). It was further assumed that community optometrists would detect conversion to OAG if the patient progressed to moderate or severe glaucoma, or visual impairment.

(2) Frequency of clinical visits

The frequency of visits in the model depends on (a) disease status, (b) whether the last IOP measurement is “on target” and (c) whether a patient has been treated (only for OHT patients). During each visit, both IOP and VF were measured (Table A6). Visit frequency gradually decreased if there was no sign of disease progression and remained unchanged at a certain point. For the OHT (treated) and mild glaucoma patients, the required visit frequency was relatively low given the low risk of progression to visual impairment. For moderate or more severe stages of glaucoma patients, time interval between two visits became shorter. Untreated patients were recommended for an annual check-up.

For a patient whose IOP measured during the last clinical visit was "on target", the next visit was timed based on Table A6 below. If "off target", the length of time interval remained unchanged compared with the last interval. For example, if an (treated) OHT patient's first visit occurred 3 months ago, and the IOP was considered "on target", the next clinical visit would be 6 months after the first visit; if IOP was "off target", the next clinical visit would still be 3 months.

Table A6: frequency of optometrists or ophthalmologist visits by treatment (in months)

**Visit number**	**Monitoring intervals (treated patients)**	**Monitoring intervals (untreated patients)**
**OHT patients**		
1	3	12
2	6	12
3 or more	12	12
**OAG patients (mild glaucoma)**		
1	3	
2	6	
3 or more	12	
**OAG patients (moderate or severe glaucoma)**		
1	1	
2	3	
3 or more	6	

6. The unit cost and utility values

The unit cost for a visit to the NHS ophthalmology services was obtained from the NHS reference cost. Following Burr et al. (2012),[1] this unit cost was assumed to include the IOP test only, whilst the unit cost for both the IOP and VF tests was assumed to be twice the cost of IOP test given the time needed to complete the visit. The unit cost for IOP and VF tests under community optometrist settings was assumed to be equal to an NHS sight test fee.[1] Following the same logic, the fee for the IOP-only test was halved. Medications and surgical treatments were valued using national unit cost sources (Table 2). We used the EQ-5D to value quality of life for each disease state in the model (i.e., OHT, mild, moderate, severe glaucoma and visual impairment) based on a valuation study of an OAG population from the UK.[15] Given small differences in visual damage between OHT and mild OAG, the utility scores for these two states were assumed to be the same.[1] We assumed no reductions in quality of life due

to treatment side effects based on the notion that side effects would either be mild for a very short period of time, or would trigger a treatment change.

The unit cost for the SLT was extracted from the LiGHT trial, which compared the clinical and economic effectiveness of using SLT as a first-line treatment for OHT and glaucoma patients with traditional eyedrops as first line drugs.[7] The trial has led to a change in NICE guidelines, in which SLT is now recommended as the first-line treatment for newly diagnosed OHT and OAG patients.[16]

Supplementary Material A2: model validation and calibration

The model has been carefully validated based on the internal (EMRs) dataset used and a number of external data sources, and several calibrations have been made. The validation tasks conducted were: (1) validating glaucoma progression rate and time-to-progression using the results from the EMGT study;[5] (2) validating time-to-conversion using the EMRs and (3) validating the proportion of “on target” patients in each medication using the results from the LiGHT trail.[7]

(1) Task 1: validating progression rate and time-to-progression

The EMGT study is a 6-year trial in which the effects of medication in reducing IOP in early untreated OAG were investigated . The results suggest that the average progression rate for treated patients is –0.03 dB per month (i.e., -0.36dB/year). To compare with this result, we aligned our model with the baseline characteristics of patients in the EMGT study (i.e., baseline IOP=20.6 (SD=4.1) assuming a gamma distribution; baseline MD = -5 (SD=3.7) assuming a gamma distribution; no trabeculotomy is allowed). Our validation results suggested that the average progression rate is -0.27dB/year, significantly lower than the one in the EMGT study.

Relevant calibration conducted: Given the slower glaucoma progression of the patients in this model compared with the EMGT study, we changed the method to calculate the progression rate by dropping the condition that the annual glaucoma progression rate (i.e., mean deviations) is allowed to be equal to zero when IOP <13 mmHg, originally specified in van Gestel (2012)[6] . The model was rerun and the

results showed that the calibrated annual rate of progression is -0.33dB/year, similar to the one in the EMGT study.

We further validated the model by comparing the time-to-progression estimates (closedly linked to the progression rate) in this model with the findings from a systematic review study in which time of progressing to different stages of glaucoma were calculated from multiple sources.[17] Again, we align the baseline setting of the testing model with the one in the systematic review study (i.e., baseline MD = -4(SD=2) dB per year assuming a gamma distribution for comparing the time-to-progression estimates of the two models from mild to moderate, MD = -6.02(SD=2) dB per year for comparing time-to-progression from moderate to severe, and MD = -12.02(SD=2) dB per year for comparing time-to-progression from severe to visual impairment. All the patients started with mild glaucoma when comparing time-to-progression estimates from mild to glaucoma, and in a similar fashion, started with moderate glaucoma when comparing time-to-progression from moderate to severe and started with severe glaucoma when comparing time-to-progression from severe to visual impairment. The model was run for lifetime). The results presented in Table A7 suggest that the progression in this model is generally slightly slower than those reported in Burr et al. (2007),[17] but the differences are within a acceptable range.

Table A7: Results for validation task 1

	**Average progression rate (dB/year) – being calibration**	**Average progression rate (dB/year) – after calibration**	**Time-to-progression (mild to moderate)**	**Time-to-progression (moderate to severe)**	**Time-to-progression (severe to visual impairment)**
**EMGT study**	-0.36	/	/	/	/
**Burr et al. (2007)**	-0.27	-0.33	5	14	16
**This model**	/	/	8	16	19

(2) Task 2: validating the proportion of “on target” patients in each medication

The LiGHT study is a 3-year trial in which the clinical effectiveness of using SLT instead of eyedrops as a first-line treatment for the newly diagnosed OHT and OAG patients was investigated.[7] The HTA report (Table 11 in page 135) showed the proportion of “on-target” patients after first-line and second-line medications, which can be used to verify the results in this study.[7] The first step of validation was aligning our testing model with the baseline characteristics of patients in the LiGHT study (i.e., (a) consistent baseline variables: baseline IOP = 24.4(SD=5); baseline MD = -3(SD=3.6); Baseline age = 62.7 (SD=11.6). (b) consistent initial proportion of patients in each state: 29.7% in OHT; 52.3% in mild OAG; 12.4% in moderate OAG; 5.6% in severe OAG. (c) all patients were treated at the beginning). The validation results in Table A8 suggested that the proportions in first or second-line treatment at each year were much smaller than those found in the LiGHT study.

Relevant calibration conducted: An adherence rate of 75% for the OHT patients had been considered initially based on Burr et al. (2012),[1] yet later calibrated to 100% based on the LiGHT trial results. 100% adherence rate was also a reasonable assumption as the distributions of medication effectiveness used in the model already incorporated the low effectiveness because of non-adherence.

Validation results after calibration:

It can be seen from Table A8 that the proportions of patients who stayed on first or second-line drugs after calibration were closer to those from the LiGHT trial. For example, the proportion of on-target patients after first-line medication in the LiGHT trial is 89.6% and 86% in our model assuming a 100% adherence rate. We also observed that the actual proportions in the LiGHT trial dropped faster than those in our model. The gaps may be attributed to the following factors: (a) The distributions of baseline IOP for different glaucomatous stages can be different in the trial, but the authors only reported an overall distribution for the OAG patients; (b) the rules of treatment escalation were different between the trial and this model. For example, the LiGHT trial allowed for re-adjustment of IOP target depending on the control of IOP, which was not specified in this model; (c) the IOP targets

used were different – the targets in the LiGHT were generally more stringent to achieve; (d) the results reported in the LiGHT trial were at eye level instead of patient level. Given all the differences mentioned above, the calibration for this task was only based on the results of first visit and at 12 months. However, results of longer time were reported for transparency.

Table A8: Results for validation task 2

	Firstline medication	Second-line or further medication	Source
**First visit**			
LiGHT trial	89.6%	/	Gazzard et al. (2019)[7]
This model (75% adherence rate)	65%		
This model (100% adherence rate)	86%		
**At 12 months**			
LiGHT trial	82.2%	13.1%	Gazzard et al. (2019)[7]
This model (75% adherence rate)	72%	25%	
This model (100% adherence rate)	87%	10%	
**At 24 months**			
LiGHT trial	71.5%	20.4%	Gazzard et al. (2019)[7]
This model (75% adherence rate)	69%	26.5%	
This model (100% adherence rate)	83%	13%	
**At 36 months**			
LiGHT trial	64.6%	25.6%	Gazzard et al. (2019)[7]
This model (75% adherence rate)	66%	27.5%	
This model (100% adherence rate)	79%	13.2%	
**At 72 months**			
LiGHT trial	42.6%	27.6%	Gazzard et al. (2023)[18]
This model (75% adherence rate)	56%	33%	
This model (100% adherence rate)	67%	19%	

(3) Task 3: validating time-to-conversion

Using the original time-to-conversion equation from van Gestel (2012)[6] resulted in a 5-year conversion rate of 10.9%, which differed from the observed conversion rate (i.e., 16.9%) from the EMRs sample (i.e., the individual sampling dataset we used in this study), suggesting an overall higher risk profile of this sample compared with the OHTS dataset referenced by van Gestel (2012).[6]

Relevant calibration conducted: In the calculation of time-to-conversion, hazard ratio for the referenced population was increased from 0.02 to 0.03, proportional to the higher risk found in the EMR sample vs the rate found in van Gestel (2012)'s study, to reflect the higher risk of the cohort used in the model.

Validation results after calibration:

The estimated conversion rate after calibration was 15.3%, which was closer to the observed conversion rate of the EMRs sample.

Supplementary Material A3: sensitivity analyses

(1) One-way sensitivity analysis

A number of parameter inputs were tested using one-way sensitivity analysis: (a) the threshold of treatment decision regarding the RP strategy; (b) medication and monitoring costs; (c) adherence rate to medication. We expect that a higher risk threshold for the RP strategy may change the CE results, Therefore, we varied the value from 6% (base case) to 20%. Higher medication or monitoring cost could increase the difference of total cost between the RP and SC strategies, which may change the CE results. As the unit costs of the pharmaceutical brands used in the base-case analysis were already the highest NHS indicative prices, we increased the unit costs of PGA and PGA&BB from +0% (base case) to 50%. Similarly, we increased the unit costs of primary care and secondary care tests (IOP only and full tests) by up to 50%, as no alternative source of unit costs can be used. Adherence rate can affect the proportion of on-target IOPs, and subsequently affect the QALYs results. We decreased the

adherence rate from 100% (base case) to 75% (used in Burr et al. (2012)). The results of the one-way sensitivity analysis can be found in Table A9.

Table A9: one-way sensitivity analysis results

Variable value	Strategy	Cost	Inc. cost	Eff	Inc. eff	ICER
Risk threshold						
0.06 (base case)	SC	£4,659		10.9006	0.0000	
0.06 (base case)	RP	£4,931	£272	10.9231	0.0225	£12,100
0.08	SC	£4,659		10.9006	0.0000	
0.08	RP	£4,918	£259	10.9211	0.0205	£12,632
0.1	SC	£4,659		10.9006	0.0000	
0.1	RP	£4,889	£230	10.9156	0.0150	£15,342
0.12	SC	£4,659		10.9006	0.0000	
0.12	RP	£4,838	£179	10.9088	0.0082	£21,896
0.14	SC	£4,659		10.9006	0.0000	
0.14	RP	£4,793	£134	10.9003	-0.0003	-£480,706
0.16	SC	£4,659		10.9006	0.0000	
0.16	RP	£4,747	£88	10.8913	-0.0092	-£9,483
0.18	SC	£4,659		10.9006	0.0000	
0.18	RP	£4,695	£36	10.8835	-0.0171	-£2,117
0.2	RP	£4,649		10.8766	0.0000	
0.2	SC	£4,659	£10	10.9006	0.0240	£414
Adherence						
0.75	SC	£4,851		10.8964	0.0000	
0.75	RP	£5,211	£360	10.9179	0.0215	£16,729
0.8	SC	£4,826		10.8970	0.0000	
0.8	RP	£5,159	£333	10.9187	0.0217	£15,348
0.85	SC	£4,773		10.8982	0.0000	
0.85	RP	£5,087	£314	10.9201	0.0219	£14,330
0.9	SC	£4,725		10.8989	0.0000	
0.9	RP	£5,027	£301	10.9210	0.0220	£13,673
0.95	SC	£4,686		10.8998	0.0000	
0.95	RP	£4,979	£292	10.9220	0.0222	£13,159
1 (base case)	SC	£4,659		10.9006	0.0000	
1 (base case)	RP	£4,931	£272	10.9231	0.0225	£12,100
Cost of PGA						
144.04 (base case)	SC	£4,659		10.9006	0.0000	
144.04 (base case)	RP	£4,931	£272	10.9231	0.0225	£12,100
158.444 (+ 10%)	SC	£4,728		10.9006	0.0000	
158.444 (+ 10%)	RP	£5,027	£299	10.9231	0.0225	£13,296
172.848 (+ 20%)	SC	£4,796		10.9006	0.0000	
172.848 (+ 20%)	RP	£5,123	£326	10.9231	0.0225	£14,491
187.252 (+ 30%)	SC	£4,865		10.9006	0.0000	

Table A9: continued

187.252 (+ 30%)	RP	£5,218	£353	10.9231	0.0225	£15,686
201.656 (+ 40%)	SC	£4,934		10.9006	0.0000	
201.656 (+ 40%)	RP	£5,314	£380	10.9231	0.0225	£16,881
216.06 (+ 50%)	SC	£5,002		10.9006	0.0000	
216.06 (+ 50%)	RP	£5,409	£407	10.9231	0.0225	£18,076
Cost of PGA and BB						
165.27 (base case)	SC	£4,659		10.9006	0.0000	
165.27 (base case)	RP	£4,931	£272	10.9231	0.0225	£12,100
181.797 (+ 10%)	SC	£4,703		10.9006	0.0000	
181.797 (+ 10%)	RP	£4,992	£288	10.9231	0.0225	£12,793
198.324 (+ 20%)	SC	£4,748		10.9006	0.0000	
198.324 (+ 20%)	RP	£5,052	£304	10.9231	0.0225	£13,485
214.851 (+ 30%)	SC	£4,792		10.9006	0.0000	
214.851 (+ 30%)	RP	£5,112	£319	10.9231	0.0225	£14,177
231.378 (+ 40%)	SC	£4,837		10.9006	0.0000	
231.378 (+ 40%)	RP	£5,172	£335	10.9231	0.0225	£14,869
247.905 (+ 50%)	SC	£4,881		10.9006	0.0000	
247.905 (+ 50%)	RP	£5,232	£350	10.9231	0.0225	£15,561
Cost of secondary care full test						
294 (base case)	SC	£4,659		10.9006	0.0000	
294 (base case)	RP	£4,931	£272	10.9231	0.0225	£12,100
323.4 (+ 10%)	SC	£4,937		10.9006	0.0000	
323.4 (+ 10%)	RP	£5,191	£253	10.9231	0.0225	£11,252
352.8 (+ 20%)	SC	£5,216		10.9006	0.0000	
352.8 (+ 20%)	RP	£5,450	£234	10.9231	0.0225	£10,404
382.2 (+ 30%)	SC	£5,494		10.9006	0.0000	
382.2 (+ 30%)	RP	£5,709	£215	10.9231	0.0225	£9,555
411.6 (+ 40%)	SC	£5,772		10.9006	0.0000	
411.6 (+ 40%)	RP	£5,968	£196	10.9231	0.0225	£8,707
441 (+ 50%)	SC	£6,051		10.9006	0.0000	
441 (+ 50%)	RP	£6,228	£177	10.9231	0.0225	£7,858
Cost of secondary care IOP-only test						
147 (base case)	SC	£4,659		10.9006	0.0000	
147 (base case)	RP	£4,931	£272	10.9231	0.0225	£12,100
161.7 (+ 10%)	SC	£4,679		10.9006	0.0000	
161.7 (+ 10%)	RP	£4,949	£271	10.9231	0.0225	£12,014
176.4 (+ 20%)	SC	£4,699		10.9006	0.0000	
176.4 (+ 20%)	RP	£4,967	£269	10.9231	0.0225	£11,928
191.1 (+ 30%)	SC	£4,718		10.9006	0.0000	
191.1 (+ 30%)	RP	£4,985	£267	10.9231	0.0225	£11,842
205.8 (+ 40%)	SC	£4,738		10.9006	0.0000	
205.8 (+ 40%)	RP	£5,003	£265	10.9231	0.0225	£11,756
220.5 (+ 50%)	SC	£4,758		10.9006	0.0000	

Table A9: continued

220.5 (+ 50%)	RP	£5,021	£263	10.9231	0.0225	£11,670
Cost of primary care full test						
22.26 (base case)	SC	£4,659		10.9006	0.0000	
22.26 (base case)	RP	£4,931	£272	10.9231	0.0225	£12,100
24.486 (+ 10%)	SC	£4,685		10.9006	0.0000	
24.486 (+ 10%)	RP	£4,962	£277	10.9231	0.0225	£12,308
26.712 (+ 20%)	SC	£4,711		10.9006	0.0000	
26.712 (+ 20%)	RP	£4,992	£282	10.9231	0.0225	£12,515
28.938 (+ 30%)	SC	£4,736		10.9006	0.0000	
28.938 (+ 30%)	RP	£5,023	£286	10.9231	0.0225	£12,722
31.164 (+ 40%)	SC	£4,762		10.9006	0.0000	
31.164 (+ 40%)	RP	£5,053	£291	10.9231	0.0225	£12,930
33.39 (+ 50%)	SC	£4,788		10.9006	0.0000	
33.39 (+ 50%)	RP	£5,084	£296	10.9231	0.0225	£13,137
Cost of primary care IOP-only test						
11.13 (base case)	SC	£4,659		10.9006	0.0000	
11.13 (base case)	RP	£4,931	£272	10.9231	0.0225	£12,100
12.243 (+ 10%)	SC	£4,659		10.9006	0.0000	
12.243 (+ 10%)	RP	£4,932	£273	10.9231	0.0225	£12,104
13.356 (+ 20%)	SC	£4,660		10.9006	0.0000	
13.356 (+ 20%)	RP	£4,932	£273	10.9231	0.0225	£12,107
14.469 (+ 30%)	SC	£4,660		10.9006	0.0000	
14.469 (+ 30%)	RP	£4,933	£273	10.9231	0.0225	£12,110
15.582 (+ 40%)	SC	£4,660		10.9006	0.0000	
15.582 (+ 40%)	RP	£4,933	£273	10.9231	0.0225	£12,114
16.695 (+ 50%)	SC	£4,661		10.9006	0.0000	
16.695 (+ 50%)	RP	£4,934	£273	10.9231	0.0225	£12,117

(2) probabilistic sensitivity analysis

A number of distributions were generated to describe the second-order uncertainty around the mean parameters for the utility, costs and treatment effectiveness. These distributions were then used in the probabilistic sensitivity analysis. The parameter inputs are presented in Table A10.

Table A10: Parameters and sources for probabilistic sensitivity analysis

		Parameter	Distribution	Data source
**Treatment effectiveness**				
	PGAs (Latanoprost)	Mean: 29.5% (base case)<br>SD: 1%	Beta	SD Based on the 95% confidence interval in Valk et al. (2005)[12]
	PGAs & BB (Latanoprost & Timolol) as second-line treatment (additional effectiveness compared with Latanoprost)	Mean: 14.1% (base case)<br>SD: 3%	Beta	SD Based on the 95% confidence interval in Webers et al. (2008)[13]
	SLT (additional effectiveness compared with PGAs)	Mean: 0.312 (base case)<br>SD: 0.015	Beta	SD Based on the 95% confidence interval in Chi et al. (2020)[8]
**Costs for treatments**				
	Latanoprost	Min=-10%<br>Likeliest =mean (base case)<br>Max=+10%	Triangular	Assumption
	Latanoprost &Timolol	Min=-10%<br>Likeliest =mean (base case)<br>Max=+10%	Triangular	Assumption
	SLT	From £96 to £151	Uniform distribution	LiGHT study (Gazzard et al, 2019)[7]
	Trabeculotomy	Mean=£1,706<br>SD=£1,302	Empirical distribution from all types of cares (e.g., elective, non-elective)	NHS reference costs (2021-2022); Glaucoma surgical procedures (HRGs code: BZ92B)
**Cost for monitoring**				
	The assumption between the price of IOP-only test and full test	Mean: 2 (times)<br>SD: 0.5	Normal distribution	Assumption

Table A10: continued

**Utility**			
Utility for mild OAG	Mean=0.8015 (base case)<br>SD=0.01	Beta	Burr, Kilonzo, et al. (2007)[15]
Utility for moderate OAG	Mean=0.7471 (base case)<br>SD=0.01	Beta	Burr, Kilonzo, et al. (2007)[15]
Utility for severe OAG	Mean=0.7133 (base case)<br>SD=0.01	Beta	Burr, Kilonzo, et al. (2007)[15]
Utility for visual impairment	Utility for severe OAG*multiplier distribution (u= -0.31029; σ= 0.16631)	Lognormal (multiplier distribution)	Burr, Mowatt, Hernández, et al. (2007)[19]

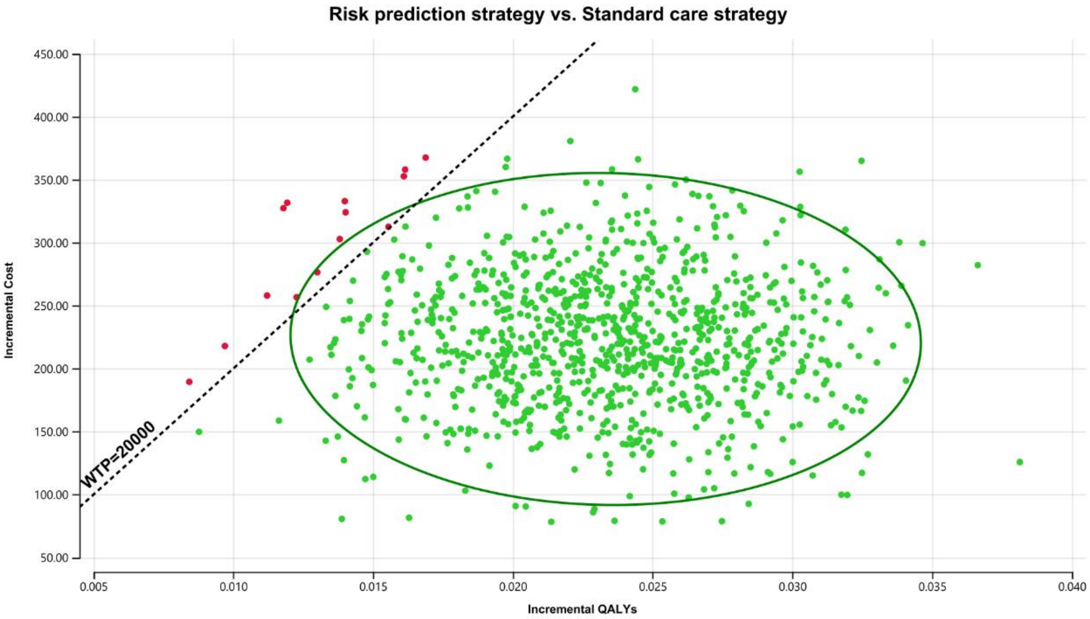

Figure A2: Cost-effectiveness Scatterplots

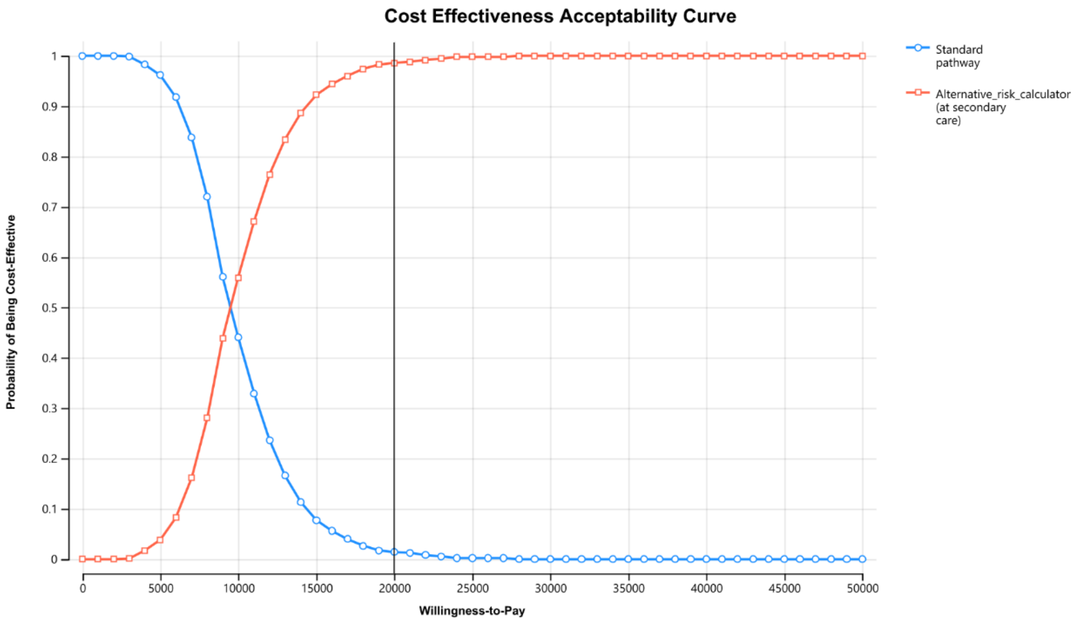

Figure A3: Cost Effectiveness Acceptability Curve

References

1. Burr JM, Botello-Pinzon P, Takwoingi Y, et al. Surveillance for ocular hypertension: an evidence synthesis and economic evaluation. *Health Technol Assess*. 2012;16(29):1-266. doi:10.3310/HTA16290

2. Gordon MO, Torri V, Miglior S, et al. Validated prediction model for the development of primary open-angle glaucoma in individuals with ocular hypertension. *Ophthalmology*. 2007;114(1):10-19.e2. doi:10.1016/J.OPHTHA.2006.08.031

3. Kass MA, Gordon MO, Gao F, et al. Delaying treatment of ocular hypertension: the ocular hypertension treatment study. *Arch Ophthalmol*. 2010;128(3):276-287. doi:10.1001/ARCHOPHTHALMOL.2010.20

4. Van Gestel A, Severens JL, Webers CAB, Beckers HJM, Jansonius NM, Schouten JSAG. Modeling complex treatment strategies: construction and validation of a discrete event simulation model for glaucoma. *Value Health*. 2010;13(4):358-367. doi:10.1111/J.1524-4733.2009.00678.X

5. Heijl A, Leske MC, Bengtsson B, Hyman L, Bengtsson B, Hussein M. Reduction of Intraocular Pressure and Glaucoma Progression: Results From the Early Manifest Glaucoma Trial. *Archives of Ophthalmology*. 2002;120(10):1268-1279. doi:10.1001/ARCHOPHT.120.10.1268

6. Gestel A van. Glaucoma management : economic evaluations based on a patient level simulation model. Published online January 1, 2012. doi:10.26481/DIS.20121005AG

7. Gazzard G, Konstantakopoulou E, Garway-Heath D, et al. Selective laser trabeculoplasty versus drops for newly diagnosed ocular hypertension and glaucoma: the LiGHT RCT. *Health Technol Assess (Rockv)*. 2019;23(31):1-101. doi:10.3310/HTA23310

8. Chi SC, Kang YN, Hwang DK, Liu CJL. Selective laser trabeculoplasty versus medication for open-angle glaucoma: systematic review and meta-analysis of randomised clinical trials. *Br J Ophthalmol*. 2020;104(11):1500-1507. doi:10.1136/BJOPHTHALMOL-2019-315613

9. Landers J, Martin K, Sarkies N, Bourne R, Watson P. A twenty-year follow-up study of trabeculectomy: risk factors and outcomes. *Ophthalmology*. 2012;119(4):694-702. doi:10.1016/J.OPHTHA.2011.09.043

10. Kirwan JF, Lockwood AJ, Shah P, et al. Trabeculectomy in the 21st century: a multicenter analysis. *Ophthalmology*. 2013;120(12):2532-2539. doi:10.1016/J.OPHTHA.2013.07.049

11. Crabb DP, Russell RA, Malik R, et al. Frequency of visual field testing when monitoring patients newly diagnosed with glaucoma: mixed methods and modelling. *Health Services and Delivery Research*. 2014;2(27):1-102. doi:10.3310/HSDR02270

12. Van Der Valk R, Webers CAB, Schouten JSAG, Zeegers MP, Hendrikse F, Prins MH. Intraocular pressure-lowering effects of all commonly used glaucoma drugs: a meta-analysis of randomized clinical trials. *Ophthalmology*. 2005;112(7):1177-1185. doi:10.1016/J.OPHTHA.2005.01.042

13. Webers CAB, Beckers HJM, Nuijts RMMA, Schouten JSAG. Pharmacological management of primary open-angle glaucoma: second-line options and beyond. *Drugs Aging*. 2008;25(9):729-759. doi:10.2165/00002512-200825090-00002

14. Azuara-Blanco A, Burr J, Thomas R, Maclennan G, McPherson S. The accuracy of accredited glaucoma optometrists in the diagnosis and treatment recommendation for glaucoma. *Br J Ophthalmol*. 2007;91(12):1639-1643. doi:10.1136/BJO.2007.119628

15. Burr JM, Kilonzo M, Vale L, Ryan M. Developing a preference-based Glaucoma Utility Index using a discrete choice experiment. *Optom Vis Sci*. 2007;84(8):797-808. doi:10.1097/OPX.0B013E3181339F30

16. National Institute for Health and Care Excellence. NICE: Guidance on Glaucoma: Diagnosis and Management of Chronic Open Angle Glaucoma and Ocular Hypertension. https://www.nice.org.uk/guidance/NG81 (accessed 20 April 2022).

17. Burr JM, Mowatt G, Hernández R, et al. The clinical effectiveness and cost-effectiveness of screening for open angle glaucoma: a systematic review and economic evaluation HTA Health Technology Assessment NHS R&D HTA Programme www.hta.ac.uk. *Health Technol Assess (Rockv)*. 2007;11(41). http://www.hta.ac.uk

18. Gazzard G, Konstantakopoulou E, Garway-Heath D, et al. Laser in Glaucoma and Ocular Hypertension (LiGHT) Trial: Six-Year Results of Primary Selective Laser Trabeculoplasty versus Eye Drops for the Treatment of Glaucoma and Ocular Hypertension. *Ophthalmology*. 2023;130(2):139-151. doi:10.1016/J.OPHTHA.2022.09.009

19. Burr JM, Mowatt G, Hernández R, et al. The clinical effectiveness and cost-effectiveness of screening for open angle glaucoma: a systematic review and economic evaluation. *Health Technol Assess*. 2007;11(41). doi:10.3310/HTA11410
